# Finding an apprenticeship: hidden curriculum and social consequences

**DOI:** 10.3389/fpsyg.2015.01441

**Published:** 2015-09-30

**Authors:** Gaële Goastellec, Guillaume Ruiz

**Affiliations:** ^1^OSPS, Institute of Social Sciences, Faculty of Political and Social Sciences, University of LausanneLausanne, Switzerland; ^2^LABEDUC, Institute of Social Sciences, Faculty of Political and Social Sciences, University of LausanneLausanne, Switzerland

**Keywords:** apprenticeship, recruitment, school streams, inequalities, autonomy, subject

## Abstract

In Switzerland, the majority of students are oriented toward professional training after compulsory schooling. At this stage, one of the biggest challenges for them is to find an apprenticeship position. Matching supply and demand is a complex process that not only excludes some students from having direct access to professional training but also forces them to make early choices regarding their future sector of employment. So, how does one find an apprenticeship? And what do the students’ descriptions of their search for apprenticeships reveal about the institutional determinants of social inequalities at play in the system? Based on 29 interviews conducted in 2014 with 23 apprentices and 6 recruiters in the Canton of Vaud, this article interrogates how the dimensions of educational and social trajectories combine to affect access to apprenticeships and are accentuated by recruiters using a “hidden curriculum” during the recruitment process. A hidden curriculum consists of knowledge and skills not taught by the educational institution but which appear decisive in obtaining an apprenticeship. By analyzing the contrasting experiences of students in their search for an apprenticeship, we identify four types of trajectories that explain different types of school-to-apprenticeship transitions. We show how these determinants are reinforced by the “hidden curriculum” of recruitment based on the soft skills of feeling, autonomy, anticipation, and reflexivity that are assessed in the context of recruitment interactions. The discussion section debates how the criteria that appear to be used to identify the “right apprentice” tend to (re)produce inequalities between students. This not only depends on their academic results but also on their social and cultural skills, their ability to anticipate their choices and, more widely, their ability to be a *subject* in their recruitment search. “The Subject is neither the individual, nor the self, but the work through which an individual transforms into an actor, meaning an agent able to transform his/her situation instead of reproducing it.” (Touraine, 1992, p. 476).

## Introduction

In Switzerland, once compulsory education ends, two-thirds of students are oriented toward professional training while the remaining third go on to general secondary education. For the former, the main driving force of their schooling and training orientation is their ability to find an apprenticeship when they reach the end of compulsory education (officially at the age of 15).

Based on 29 interviews (29) conducted in 2014 with 23 apprentices and 6 recruiters in the Canton of Vaud, Switzerland, this article compares the students’ descriptions of their search for an apprenticeship with recruiters’ accounts and analyses what they reveal about the institutional determinants of social inequalities at play in the system.

Indeed, educational background appears to affect access to apprenticeships. At the time when the interviewees were enrolled in the first cycle of secondary education (before 2013), the cantonal system was organized into three different educational streams at compulsory secondary school level. These educational streams were characterized by the juxtaposition of two processes. On one hand, students were selected at about 11–12 years old on the basis of their academic results and oriented in one or another stream accordingly. These three streams were thus parts considered hierarchical by the school actors, students, and families. On the other hand, each of these pathways was supposed to lead students to different educational and professional outcomes. The VSB (secondary school baccalaureate) stream trained students to enter high school and obtain a baccalaureate, a qualification granting access to all tertiary education institutions, including universities and polytechnics. These are the most prestigious higher education institutions in Switzerland and register around 22% of one age group in the Vaud Canton (Institute for Research and Pedagogic Documentation [IRDP], 2014). The VSG (secondary school general stream) was presented as an option that points students to schools of general culture or to vocational education and training and, eventually, to a limited sub-sector of tertiary education corresponding to Universities of Applied Sciences. Finally, the VSO (secondary school “options”) stream was exclusively intended to take the weakest students and prepare them for apprenticeships.

Thus, the attempt to match different forms of training with students’ academic abilities results in sending the weakest students to a professional stream, or at least to a stream in which the only educational outcome can be professional training. This also frequently leads to allocating students to one or another stream. The initial situation can be summarized as associating weak academic results with professional orientation as well as depriving most students of any kind of choice between vocational education and training and high school.

This education system became more complex in the 90’s with the development of transitional structures at the end of compulsory education (Valli, 2012). Although first designed as a temporary measure to meet the needs of a growing number of young people facing unemployment as a result of increasing difficulties in finding apprenticeships in an economic recession, they ultimately became permanent. Over a period of 6 to 12 months, these structures offer practical and school-oriented activities, internships, orientation guidance, and assistance to find an apprenticeship (DFJC, 2014). They now account for about 20% of one age group in the Vaud Canton (SCRIS, 2011) and have been attended by 5 of the 23 apprentices in our sample. This is a reasonable match with official data.

Indeed, at the end of compulsory schooling in 2010 in the Canton of Vaud, 27.7% of students had a “general maturity” high school education (supposed to lead to university studies), 10.4% had a “general culture” high school education (giving them access to applied science universities) while 24% had professional training, 21% used transitional structures, 8% were waiting for a solution, and 7% registered for a “transition year” in order to move to a higher academic stream (SCRIS, 2011).

Education pathways thus appear more heterogeneous than initially planned as the training system was initially designed to allow direct transitions between the end of compulsory schooling and upper secondary education (Amos, 2007). In practice, an increasing proportion of schooling careers are not linear. Transitional measures accommodate a large proportion of students unable to find an apprenticeship while most students are trained in a pathway that is formally presented and assumed to prepare them for an apprenticeship.

At the same time, in recent years a number of companies were unable to find applicants for the apprenticeships they offered (SEFRI, 2013). Several explanations help understand this situation. Firstly, part of the training’ companies have been known to reject some of the applications that they received after having judged them to be unsuitable (LINK, 2013). Secondly, young people are deserting a range of industries such as construction, hotel, and catering services sectors. From one point of view, “Choosing an apprenticeship in these sectors doesn’t seem to offer a real future to young people, since the possibility to operate a professional reorientation and transferring knowledge from one CFC (Federal Certificate of Capacity) to another seems rather uncertain” (Sigerist, 2003, p. 21). On the other hand, using the approach developed by Dubar (1991), one can make the hypothesis in professional socialization terms, that the process of professional insertion via an apprenticeship is more defined by a dynamic process of identity construction than stabilizing individuals in the labor market. It is likely that some jobs or, more generally, some sectors are being ignored by future apprentices partly because of the weak social recognition they are associated with.

Conversely, some apprenticeships, such as commercial employee training, are highly esteemed by young people (SEFRI, 2014). The same applies to apprenticeships linked to occupations in the field of computer sciences, the processing industry, sales, health, or social activities (LINK, 2013). The concentration of apprenticeship requests on specific types of training contributes to stiff competition between candidates, competition in which personal resources can play a significant role. This competition is also stimulated by demographic change, structural changes, apprenticeship development policies and the ability of Swiss companies to offer training. As such, the percentage of Swiss companies that are able to train young people currently amounts to 40% (SEFRI, 2014). This competitive situation can accentuate some forms of discrimination, whether they are directed toward candidates of immigrant descent (Imdorf and Seiterle, 2015) or are gender-related (Lamamra, 2011).

When dealing with increasing numbers of applications, employers tend to either favor young people from the most demanding academic curricula instead of those from curricula initially designed lead to apprenticeships (Perriard, 2005) or they favor older people thereby guaranteeing greater maturity according to them (Vanheerswynghels, 1996). Research carried out since the beginning of the 2000s shows the existence of segregation mechanisms during the search for a place at the beginning of the apprenticeship (Imdorf, 2013; Imdorf and Seiterle, 2015) or at the end when entering the labor market as a professional (Fibbi et al., 2003). Others have studied factors of success during the training period (Häfli and Schellenberg, 2009), acknowledging difficulties experienced by apprentices when building their sexual and professional identities (Duc, 2012; Lamamra, 2014), as well as reasons why some apprentices cease their training (Lamamra and Masdonati, 2009). The TREE (Transition from Education to Employment) study which was based on a longitudinal survey of a cohort of students who finished compulsory schooling in 2000, provided structural explanations by highlighting the non-linear nature of trainees’ pathways and the roles of transition solutions between schools and apprenticeships (Amos, 2007). Nevertheless, this kind of training process is insufficiently explored in relation to the place that it occupies in the Swiss training system (Cortesi and Imdorf, 2013). Processes that build access to an apprenticeship and determine the possibility of training, in particular, are still poorly understood.

The match between supply and demand is a complex process that not only excludes some students from direct access to professional training but also forces them to make early choices (or accept their lack of choices) regarding their future sector of employment. How does one find an apprenticeship? We hypothesize that social inequalities identified in previous research as characterizing access to apprenticeships can be explained not only by students’ academic and social backgrounds but by how the dimensions of educational trajectories and social backgrounds combine and are accentuated by a “hidden curriculum” used by recruiters during the recruitment process. A “hidden curriculum” can be defined as “the proportion of learning that does not appear to be programmed by the educational institution” (Perrenoud, 1993, p. 61) but which still influences the probability of obtaining an apprenticeship. In this case, this hidden curriculum thus consists of knowledge and skills that are not taught by the educational institution yet which appear decisive in obtaining an apprenticeship.

The following section clarifies the methodology used to explore this issue while the third section presents the results. Firstly, it corroborates what previous research has shown regarding the impact of educational and social determinants and identifies how these dimensions interact by comparing students’ trajectories with access to apprenticeships. Secondly, it uncovers a hidden recruitment curriculum through an in-depth analysis of recruitment criteria in the recruiting process as they emerge in the descriptions provided by students and recruiters. This is characterized by the importance recruiters give to soft skills such as “feeling,” autonomy and anticipation. Lastly, the discussion section questions the implications of such a hidden curriculum and how it promotes students who are able to present themselves as a *subject* in their recruitment search. Touraine has shown that the subject arises as a consequence of an effort produced by the individual to override social constraints. It refers to the participant’s share of liberty and implies a process of self-reflection. The subject can be subsumed by three characteristics: ability to distance oneself, reflexivity, and affecting social situations.

In the frame of our study, such an approach allows the analysis to be focused on individuals’ representations and strategies. It leads to questioning the context of apprentice recruitment as either facilitating or affecting the student’s ability to present themself as a subject. Methodologically, this assumes that interviewees are considered as “actors, and not as objects of observation.” (Touraine, 1982, p. 20)

## Materials^1^ and Methods

### Comparing Apprentices, Recruiters, and Institutional Accounts

In order to reveal the hiring practices and hidden curriculum involved, we conducted qualitative research on both apprentices and recruiters. The sample of young people interviewed (*n* = 23) comprised 11 men and 12 women aged from 16 to 25. Two of them were interviewed during their first year of apprenticeship, six of them during their second year, four of them during their third year, and one of them during his fourth year. Seven of them had completed their apprenticeships. Five of them had been through transitory measures, i.e., nearly one in four. The training programs represented in the sample include a technician, a dental assistant, two pharmacy assistants, a carpenter, two hairdressers, a civil engineering designer, eight commercial employees, two sellers, a heating installer, two booksellers, a pastry confectioner and a 3D polydesigner.

The recruiters (*n* = 6, four men and two women), or persons in charge of apprentice recruitment in the companies are either business managers, store managers, or people attached to services that specifically deal with company staff. None of the six recruiters occupied the same position in their respective professional contexts. During the recruitment process, they are assisted by others individuals in the company who intervene at different stages in the process.

Each interview was conducted in a semi-directive manner based on the comprehensive interview method (Kaufmann, 1996). After preparing an early version of two different interview grids, one for apprentices and one for recruiters, and conducting a first wave of 15 interviews at the end of March 2014, we readjusted the apprentices’ grids according to the responses we obtained. This readjustment mainly consisted in sorting the questions in order to start with the more general ones and leave more room for the apprentices to first explain what seemed important for them in their apprenticeship and, more broadly, in their schooling history. This became necessary given that the first wave of interviews lead us to acknowledge that what we initially hypothesized, i.e., the standardized tests that companies sometimes use to hire apprentices as being important, these were not central to the apprentices’ comments and this hypothesis should be abandoned. After these modifications, a second wave of 14 interviews with new interviewees was conducted at the end of April 2014. Questions were organized into three themes: beginning an apprenticeship, schooling, and family background. Each theme was firstly interrogated using a very broad question, aimed at letting the interviewee explain what seemed important to them. Follow-up questions were then asked to obtain more detailed information. In the end, the student was asked if they thought there was any important information that we did not ask about but which seemed important to them. Both grids resulted in the same information being obtained but the second version allowed more flexibility for interviewees to organize the story they wanted to tell by themselves. As for recruiters, the interview grid included five thematic sections: the company as a training space, recruitment methods, application file selection, hiring interviews, and probationary training-periods.

The choice to compare the accounts and practices of two different groups of actors, recruiters, and apprenticeship candidates is driven by our aim to shed light on the components of the hidden curriculum by comparing their perception of the process. Interviewees were selected at random. Some belonged to our students’ social environment while others were recruited in their workplace after asking them if they would agree to participate in some research. They were not paid for their participation abiding with local and disciplinary tradition. Although the sample size in itself does not allow generalizations to be made, the range of companies in which apprentices and recruiters were involved coupled with similarities in the aspects they raised despite this contextual diversity, supports some generalization. Furthermore, these generalizations were made possible by intensive analytical work carried out on the interviews. In-depth analysis “revealed the consistency in attitudes and social behaviors, by grounding them in a history or a trajectory that is both personal and collective” (Beaud, 1996, p. 234) but also by comparing recruiters and apprentices’ comments on their hiring and application practices, as well as the recurrences they raise.

The analysis was undertaken using an iterative approach in which we moved back and forth between data collection and data analysis and then between the analytical components themselves. These procedures are important to obtain quality data and produce plausible interpretations of the findings as well as reaching data saturation (Mukamurera et al., 2006).

This iterative approach involved a three-stage process to build an inter-rater agreement: “developing a coding scheme with as high a level of intercoder reliability as possible based on a sample of transcripts, (…) adjudicating the remaining coding disagreements through a negotiation among coders in an effort to establish a high level of intercoder agreement (…) and deploying that coding scheme on the full set of transcripts once acceptable levels of intercoder reliability and/or agreement have been achieved.” (Campbell et al., 2013, p. 298). These three stages were applied to a two-step analysis exercise. Firstly, a thematic analysis of the transcribed interviews was undertaken based on the following question: what dimensions in the recruitment process are identified as significant by both apprentices and recruiters? Next, using the same procedure, we undertook a life-course analysis with each interview. By considering several variables, such as sex, social origins, educational background, or the kind of help the apprentices had during their apprenticeship search process (social capital and family support), we identified four types of trajectory configurations that enabled us to build four ideal-type constructs to better understand the characteristics that help obtain an apprenticeship more or less quickly and, last but not least, to get into the chosen professional field, or not. Indeed, for different reasons such as inadequate school exam results, several interviewees could not get an apprenticeship in their desired professional field.

Once the two analysis exercises were completed, we went back to the interviewees’ transcripts and proceeded to identify of all excerpts illustrating the various themes acknowledged as being central to the inter-rater agreement. The excerpts presented in the results section have been selected on the basis of their being representative, i.e., they illustrate dimensions that are transversal to various interview contents. The translation of the interview sequences was developed by focusing on the meaning given by the respondent and not through a word-for-word translation to guarantee semantic correspondence.

## Results

### Revealing the Hidden Curriculum of Access to Apprenticeships

How does one find an apprenticeship? This question is divided into two sub-questions that structure this section. These are, what are the educational and social characteristics that affect access to apprenticeships and how do these dimensions interact? Which dimensions are assessed in the recruitment process through interactions, once shortlisting based on academic records is over?

#### Access to apprenticeships: various trajectories, diverse outcomes

The students interviewed were all apprentices, but had experienced very different transitions to apprenticeship depending on their previous educational streams, atypical schooling trajectories, family support and social capital.

##### Educational streams

Our research corroborates other studies on school-to-work transitions. Firstly, like Haeberlin et al. (2004), the educational pathway followed appeared to influence the quality of the transition, especially the time searching for an apprenticeship. The less prestigious the school career, the longer the length of the search. Moreover, although all of interviewees from the VSB stream, without exception, found an apprenticeship in their intended professional field, a majority of interviewees from the VSO stream (three out of five) could not obtain an apprenticeship in the fields that they wanted. This supports an observation previously made by Rastoldo (2006) in a study undertaken in the Canton of Geneva. Indeed, the recruiter’s responsibility goes beyond hiring an apprentice who can satisfy the company’s criterions, as it integrates the educational dimension. Employing someone means giving access to academic training for the apprentice, and their expectations may be different and higher than those in the company. As the success of the apprenticeship is based on the apprentice’s ability to manage both academic training and professional integration, the recruiter has to assess the two dimensions simultaneously. In this regard, the previous schooling stream appears to play an important role in identifying the “right” apprentices, and students seemed to be very aware of the labeling process at play in schooling streams:

“*When I applied for this apprentice position, it was absolutely not what I wanted to do (retail trade apprenticeship), but I was pretty sure they would choose me as I came from the VSG schooling stream so I was more…, I was a bit better than the others.*”

(Apprentice, commercial employee, female, 20 years old)

“*The fact that you have a high school diploma helps them to know that you have general knowledge, so they don’t ask for more.*”

Apprentice, bookseller, 21 years old)

“*When hiring me, I’m sure that the boss wasn’t taking many risks at the education level, results and so on, because he knew I went to high school and university. On the other hand, for someone who comes out from VSO, this will be… maybe a higher risk for them, for you to succeed in your apprenticeship so that they don’t hire someone just… to look nice.” (…) “Now, if you come out of a VSO or VSG stream, well, forget it, it still tough.*”

Apprentice, dental assistant, female, 23 years old)

And indeed, when this dental assistant described the way she was hired, she underlined the fact that she neither had to write a letter of motivation nor to go through a formal interview.

##### Atypical schooling trajectories: a possible asset

As studies have already shown (Amos, 2007; Lamamra and Masdonati, 2009), atypical trajectories, in the sense that they do not embed in the linear model envisaged by the Swiss training system, are far from being exceptions. Still, students tended to perceive atypical education trajectories, and especially transitional structures, as stigmatizing and a possible handicap for pursuing their education. Among the young people we interviewed, five apprentices attended a transitional structure and six others initially went to high school (four of them completed it), meaning that almost half of our sample can be characterized by a non-linear trajectory. Although recruiters did not value all atypical trajectories, some of them, by contrast, appeared to be sought after.

In the case of apprenticeship access, in the majority of cases, a non-linear trajectory, providing that the transitional period had been a success (completion of other studies, a language course abroad, commenced a first apprenticeship and then changed for another, or completion of a transitional measure), provided a certain advantage. Going through a transitional measure could thus be turned into an advantage, as the following interview extract shows:

“*For a young student coming out of VSO, I have to say I am a bit reticent, but for a VSO student who does an OPTI*^2^ *year with good results, there’s no problem because they have gained an additional year of maturity. I really do think everything depends on maturity.*”

Recruiter, human resources manager, insurance company, female)

Furthermore, having followed other post-compulsory forms of education also appeared to provide some advantage. Among the six young people who went to high school before starting to look for an apprenticeship, five found a post within less than 2 months (only one had been looking for a placement for 3 months before finding one). The applicants without a high school education, that is to say the majority of them, have been looking for a placement for more than 4 months (four of them have been looking for 6 months or more). For example, an apprentice working on heating design projects who studied at high school for just a few years without completing it, mentioned that the company he applied to did not ask him for anything other than his CV and his highest school marks. A single interview was sufficient to be hired. Those profiles thus seemed to be particularly sought after by a certain type of employer as shown by the following extract. We can hypothesize that these profiles raise more interest in small companies where the apprentices are more likely to be rapidly subjected to performance imperatives:

“* I think in the kinds of activities we’re dealing with require a certain kind of maturity, I mean…we are not necessarily more mature at 18 than at 16 years old, but we estimate that people who are older than 18 have already acquired a kind of maturity and have often followed an upper-level school career, whether they’ve completed high school or decided to stop it for several reasons, such as, well, failure or a lack of motivation, but they are still often people who have got the ability to obtain a high school degree.*”

Recruiter and trainer, Carpentry Company, male)

Moreover, although transitional measures increased the chances to obtain an apprenticeship position, they did not appear to increase the probability of accessing an apprenticeship in the desired sector. In this regard, high school education made a difference as only one former high school student told us he could not start the training he wanted to. Non-linear trajectories are also valued because they provide employers with apprentices that are a bit older, using age as a criterion. Discussing the recruitment interview, an apprentice answered:

“*I had anticipated the fact that I was older and that maybe I was more aware of the importance of this type of training.*”

Apprentice, commercial employee, female, 21 years old)

Another apprentice, following the same commercial apprenticeship, emphasized that:

“*When I finished school I was too young for an apprenticeship. I was 15, I was a bit lost so I did not even apply for one. But when I turned 17, after I went through transitory measures, I felt ready. During the recruitment interviews I put forward the fact that I was now mature enough, that I had done several training courses and that I was ready to do an apprenticeship now!*”

Apprentice, commercial employee, female, 20 years old)

A recruiter corroborated the fact that being older was more of an advantage than a constraint:

“*We assume that someone who is 35 years old has what is needed to take it upon themself to give themself the means to succeed.*”

Recruiter, Electricity Company, male)

And indeed, among the young people we interviewed, those who were 17 years old or more when they decided to start (or recommence) searching for an apprenticeship placement found a position within 2 months of looking, while those who were 15 or 16 years old experienced a longer period of searching with. None of them found a place within 3 months, but more often within 4–6 months.

##### Family support

Research has already shown the importance of family support for schooling and social trajectories (see, for example, Pourtois and Desmet, 1991; Parent and Paquin, 1994). Part of the interviewees emphasized the supportive role played by family members in their search for an apprenticeship, whether in preparing their application, learning how to manage interactions in the recruitment process or finding the motivation to finally enter an apprenticeship once they found a position:

“*I have had help from my sisters who are older and know how to do it, so they helped me with the CV and the motivation letter.*”

Apprentice, commercial employee, female, 20 years old)

“*Yes, my father coached me a lot; how I would have to behave during the internship. You have to show that you know how to work. I have a father like this. He explained to me how to shake hands, shake them energetically, things like that. Apart from that, my parents helped me quite a lot on my application, the letter and everything.*”

Apprentice, 3D Polydesigner, male, 18 years old)

“*In the beginning, I didn’t want to go there (the pharmacy where she found her apprenticeship), but my mother told me “Come on, try it! You still haven’t found something.” Just because they pushed me, I accepted. If they weren’t there, I would have surely found anything whereas it was what I wanted to do. But you see, I was 15 years old and you are not necessarily aware. You don’t realize how important it is. I was 14 when I began to search for something and 15 when I found it.*”

Apprentice, pharmacy assistant, female, 21 years old)

##### Social capital

Likewise, the interviews also showed that the parents’ social capital influenced the chances of quickly finding an apprenticeship position and in the subject area desired by the student:

“*Usually, in the company I work for, they only hire apprentices every 2 years and I just fell in the year they didn’t look for anyone. But I got a boost from the employer I knew very well, that all my family knew, and he made a proposal to the company’s director to be able to employ me as an apprentice because he could see I was motivated for this job.*”

Apprentice, civil engineering designer, male, 19 years old)

The role of the family in finding an apprenticeship was highlighted when the apprentices were asked how they found an apprenticeship:

“*It was my aunt, in fact. She was living in V. and she has been going to the same hairdresser for several years. She helped me to find this place. My aunt knew somebody so, it helped me to get this post.*”

Apprentice, hairdresser, female, 21 years old)

Asked if he was trained in his father’ company, another apprentice, answered as follows:

“*No, it was another company. But it’s through my father that I got the post*”.

Apprentice, commercial employee, male, 22 years old)

Social capital can be defined as a “collective production used socially that corresponds to the whole relationships put into place between different protagonists in the student’s transition: student, teachers, and potential employers” (Lecoutre, 2006). It represented an important asset for a quarter of our interviewees, and seemed to be mainly provided by the family. Family support also appeared to exceed solely social capital. Indeed, the parents, and to a lesser extent, the elder members of the family often provided the student with two other types of support (Monette and Fournier, 2000): emotional support (encouragement, motivation) and instrumental support (helping to write and review CV’s and covering letters). The mother appeared to be the most important resource person in the students’ accounts, which is coherent with the findings of Bourdon et al. (2012). Along with family, transitional structures were also mentioned as important. Teachers, by contrast, were rarely mentioned in the comments of apprentices interviewed.

How do these dimensions interact?

To answer this question, we first characterized what was to be explained, i.e., ease in finding an apprenticeship, measured by the number of applications submitted and the length of time that the students spent searching, and the relative freedom of choice of the apprenticeship subject area. The decision to focus on the choice of a subject area, instead of the choice to do an apprenticeship appeared more meaningful for the comparison, as some interviewees had no other option but to undertake an apprenticeship. Relative freedom of choice was considered when the students expressed the feeling of having chosen an apprenticeship in a subject area that was meaningful to them.

Explanatory dimensions were characterized as follows: we defined linear schooling trajectories as those with direct access to an apprenticeship after compulsory education. The type of academic stream refers to the three types of streams that form the secondary compulsory education system in the Canton. Family support corresponds to students declaring that members of their family were helpful in some way while they were looking for an apprenticeship. Finally, social capital corresponds to a family member using their network to search for an apprenticeship.

Comparing respondents’ characteristics in this regard highlighted the multiplicity of configurations at play in student’s trajectories. In order to reduce this complexity and make sense of the various trajectories, we opted for building up “ideal-type” constructs: “An ideal type is formed by the one-sided accentuation of one or more points of view and by the synthesis of a great many diffuse, discrete, more or less present and occasionally absent concrete individual phenomena, which are arranged according to those one-sidedly emphasized viewpoints into a unified analytical construct” (Weber, 1965, p. 181).

Four “ideal types” arose from comparing individual’s trajectories that describe the four main trajectory configurations. The first two types characterize students who were able to easily find an apprenticeship in their chosen subject area, while the last two depict students who faced difficulties in finding an apprenticeship and had to take one they did not choose or value. The first type is characterized by linear schooling trajectories in the highest or medium academic stream, family support, and social capital used to find an apprenticeship that the student values. This type appeared to mainly relate to males. The second type depicts non-linear schooling trajectories in the highest or medium academic stream, access to high school and occasionally, university followed by a failure, some family support and/or social capital. The third type portrays non-linear schooling trajectories in lower or medium academic stream, use of transition measures, little family support and/or social capital and difficulties in finding an apprenticeship. Females appeared to be the main protagonists in this ideal type. Finally, the last type involves linear schooling trajectories in the weakest academic streams with no familial support.

Comparing these four ideal types revealed that what helps in understanding apprentices’ ability to choose an apprenticeship they value was not so much the linearity of the schooling trajectory but the type of academic stream the student was assigned to.

Secondly, these ideal types showed a contrast between genders. The first ideal type depicted a trend that was specific to male students for whom an apprenticeship has long been the goal. These students were academically average to good, and they had the possibility to make other choices. The choice of an apprenticeship appeared here to be as desirable as the outcome of a socially enhanced education project. Conversely, the third ideal type portrayed a path for females with low to medium levels and little family resources to make the shift from a transitional measure to a tool that improved freedom of choice.

These four dimensions identified in previous research as affecting access to apprenticeship thus combined in distinctive configurations to influence not only the probability of access to apprenticeships but also ease in finding one and the ability to identify one that made sense for the student which was not perceived as a dead-end or a meaningless option. Nevertheless, these dimensions did not answer the whole question: they appeared to be crucial in going through the first steps of the recruitment processes, i.e., obtaining a preliminary internship or an interview for an apprenticeship position. But a ‘black box’ remains concerning the specific moment of the encounter between the candidate and the recruiter during the recruitment process. It is important to explore this ‘black box’ as it sheds additional light on the four ideal-type trajectories but also accounts for possible variations.

Once a student is accepted for a preliminary internship or obtains an interview for a position, what is at stake in the process? In order to deepen our understanding, we carried out an in-depth analysis of what the interviewees’ accounts revealed as important skills that are assessed in the face-to-face recruitment process. What is it that goes on during interactions between candidates and recruiters that influences access to apprenticeships?

#### Revealing the Hidden Curriculum of Recruitment

“*There are more and more who do not understand how important their internship is.*”

Commercial employee, male, 22 years old)

Company internships experienced by young people were one of the most important steps in the process of obtaining an apprenticeship. Interviews carried with recruiters enabled two types of internships to be distinguished, each fulfilling very specific roles.

The first one, that we will call the *orientation internship*, comes into play when young people are still in compulsory schooling, often between the years preceding their last year of compulsory schooling (when students are between 14 and 16 years old). These internships were either completed during school holidays or during school hours and geared to providing an opportunity to spend a few days in a company to discover “the professional reality.” They contributed to helping young people shape their training choices.

In a second phase, occurring during the recruitment process, a second type of internship at play is the *selection internship*. Companies assemble a number of young people whose applications have been shortlisted. With one exception, every student and recruiter in our sample mentioned this first step in the recruitment process as a fundamental condition without which no contracts would have been signed. This internship lasted from 3 days to 1 week, under the watchful eye of one or several colleagues. Many applicants were then assessed *in situ*. Assessing knowledge was not a major issue in this process for the employers as the applicants had already been judged on their academic records and/or through different internal or external examinations to the company. However, soft skills were especially assessed.

##### Soft skills

“*During the entire recruitment process, it is…we do try to see…if they also have soft skills. (…) Especially, soft-skills with the other apprentices. That’s what we can see a lot. Young people who are chatting, chatting, chatting, who are saying lots of things that are inappropriate or young people who are very shy, who don’t create bonds with others, who don’t show any interests.*”

Recruiter, public administration, female)

Without always openly using the concept of soft skills, all the recruiters have repeatedly mentioned the importance they gave to attitudes and behavior either during recruitment interviews or internships. In a corporate environment, the concept of “soft skills” refers above all to a prescription of behavioral norms that can represent, according to Lichtenberger (1997) and Ségal (2006), the behavioral, relational, methodological, transversal, and generic competences, as well as responsibility taking in different contexts. Thus, being able to meet those criteria, which can change according to the type of world^3^ established in different companies (Boltanski and Thévenot, 1991), seems to influence, as Perret-Clermont and Zittoun (2002) have already shown, the chances of obtaining an apprenticeship, and more generally, a smooth school-to-work transition. The statements of some of our recruiters clearly echoed those observations and grasped the role given to certain kinds of soft skill components during the internship:

“*Well, for the young person, we will immediately see if they are shy, if they have a kind of greed, or openness, and in their speech, we will immediately see, I mean notice at least, education and respect. There are very simple things. So there’s the speech, and there’s the look that influences it a lot as well.*”

Recruiter, shop manager, large-scale distribution, male)

In addition to certain kinds of relational abilities, a type of language coupled with some respect for several esthetic codes was generally expected. The following extract is even more explicit insight into the importance of a specific type of “soft skill.” Indeed, the trainer considered it necessary to do some “cultural work” on the apprentice insofar as the apprentice must go through professional cultural integration, which requires a an existing propensity, or according to him, some “predispositions”:

“*There is some real cultural work to be done before this person can be sent to visit customers on their own 1 day. It’s something I really like to do if the person shows some predispositions and a positive attitude toward it…, at least those things I am looking for consist of being able to quickly send someone to visit customers alone to take measurements for minor matters, like taking a sequence of photos, for example. So I want to trust this person completely and this is obviously the first attitude I am waiting for and can feel. It is not so much a movie casting, but, well, it is the case to some extent.*”

Recruiter and trainer, Carpentry Company, male)

##### “Feeling”

“*We have to find an apprentice that fits in, but they have to find a company that suits them too.*”

Recruiter, Carpentry Company, male)

Soft skills assessed during internships and interviews were interlinked with other employment criteria that proved to be significant. “Feeling” was mentioned both by students and recruiters, and appeared as a central selection criterion. It refers to two components: firstly the notion of “intuition,” usually defined as a “form of immediate knowledge that doesn’t require reasoning” (“Petit Robert” dictionary) and secondly, according to our interviewees’ statements, to a positive impression the others give us. The expression of “elective affinities,” defined as an immediate sympathy based on shared tastes (Bourdieu, 1979), very directly influences the chances of getting the job, as the following extract shows:

“*In fact I believe the principal criterion is that I get along with the person that we can imagine… Because we are going to spend 3 years with this person, in a relatively close relationship, with some requirements and a responsibility for that person.*”

Recruiter, responsible of a cantonal office in the Canton of Vaud, female)

Similarly, the following statement highlighted how a positive feeling not only referred to something immediate and spontaneous (in this regard, the use of the expression “immediately” is eloquent), but also to what extent it proved to determine the applicant selection’ process:

“*There are applicants that we immediately feel. They bring with them a full application file with a basic-check (a standardized test) that we appreciate. The installers immediately come to us “This guy, he’s great!” When I talk to them, I immediately feel it. Well, we don’t wait. We make a contract proposal not to let go of this great person. We don’t wait until we have several applicants’ files and say, “Which one will I choose? This one was good.” When we ‘feel’ someone, we immediately offer them a contract.*”

Recruiter, Electricity Company, male)

Describing a meeting he had with a potential apprentice, a recruiter from a big retail company said:

“*When I arrived, I asked the customer service, “How is she?” And the colleague says, “Mum, I’ll let you see.” There was no joy in her words, and that told me what I wanted to know. I saw the girl on the other side, she may be very well and calm in her words, but the image that emerged was a goth style (…) I don’t see her doing the job. In any case, she does not fit in with my values.*”

Recruiter, Retail Company, female)

Still, this type of judgment can go both ways:

“*Personal presentation can play a role (…) Once, I had an apprentice who had a lot of piercings, but she had so many other qualities which I identified early on that in the end those visual aspects (…) But it’s really unusual…*”

“*I like people who have already dealt with a practical aspect of life in their own family history or in previous training courses.*”

Recruiter, Carpentry Company, male)

In the same vein, another recruiter mentioned taking on a student who was had a disability and was apparently not suited to the job, but he legitimized this by saying:

“*We are taking responsibility and you have to know. Well, look at it this way, today maybe it’s this young person who needs help and maybe tomorrow it’s me and if I have someone stretching out their hand, I may be happy. But, one should not think that you have to do it… just to help.” (…) “I try to make my own mind up, using my feelings to see if I get a ‘feel’ for the young person. I don’t know how to say it, but those are things that one can feel. With some young people, it doesn’t happen, so it can be a criterion to say no, it did not happen, I don’t feel like it.” “When you ‘feel’ someone, you offer them a contract straight away.*”

Recruiter, shop manager, large-scale distribution, male)

From the recruiter perspective, “feeling” and “elective affinities” appeared to be sensitive issues in the recruitment process, but these can’t be expressed in a general equation that would imply the promoting a specific type of behavior. “Feeling” and “elective affinities” depended on both the company’s professional organization and the recruiter’s specific expectations that were influenced by their values and how they defined their role.

From the young person’s perspective, having a good feeling about future employers and colleagues was equally important. Having a good feeling testified, in many cases, that the interview went well and that their chances to get the post were high. The following extract is revealing in this respect. Indeed, when the researcher asked the interviewee how his job interview went, he answered:

“*Yeah, I had a very good feeling. So I left the interview and I was like, yeah, that’s it! That’s it, I’ve got the placement!*”

Commercial employee, male, 22 years old)

A second year female apprentice in commerce expanded on the same comments:

“*I felt quite at ease. Well, there is always this question about virtues and shortcomings, but in the end I knew how to answer because I had prepared for that. But yes, I really felt at ease, because, as I was saying, we had a good feeling. So, after that you are not stressed anymore.*”

Apprentice, commercial employee, female, 19 years old)

“*During the interview, they asked very specific questions about the company’s identity. They asked me if it was an issue for me to wear a uniform, if the specific way they organized the work and the relationships between people within the company was fine with me, etc. What helped me was to know about the job; it helps a lot to know what you are talking about. I also used the file I made with all my former training placement assessments made by those who were responsible for me so that they have a fresh look at me, from another company, I mean.*”

Apprentice, 3-D Polydesigner, male, 18 years old)

As with the recruiters, the apprentices’ statements revealed the roles of “feeling” and “elective affinities” between a candidate and a recruiter. They highlighted the potential flexibility of selection criteria, depending on how two visions of the world interact. This was particularly saliently put in the following interview extract from a 22 years old medical secretary apprentice and mother of a young child:

“*At first, I made the mistake in my application letter of saying I had a baby. I wanted to explain why I had an empty year in my curriculum vitae, but I should not have mentioned that (…). During the interview, they were very worried about my daughter getting sick; they asked a lot of questions about that. If she gets sick, do I have someone I can call who can take care of her, and logically, I couldn’t say yes as I only have my grandmother. I said she may take care of her but otherwise, I also have a contract with the Red Cross so I can call them, and they come to take care of her…. I felt they were really concerned about that… But when I went to this insurance company, they did not make me feel this way because amongst the people that were there at the interview, one of the women said she had a baby while she was studying, so that was possible to do. After that, it’s clear that they did not ask me questions about my family and all that, so maybe she had a family and she told herself that it was the same for me. But, it’s true that with the others we went into more detail and when I start with the details it may be a bit frightening.*”

Apprentice, commercial employee, female, 22 years old)

It is not simply the demonstrated characteristics of the candidate that affected their ability to obtain an apprenticeship but also the perception the recruiter had of how these characteristics may interfere with the professional activity.

###### “Feelings” and gender: the influence of stereotypes

Although the apprentice interviews exposed no differences between males and females in the recruitment process and its outcomes, interviews with recruiters suggested some invisible institutional barriers that shed light on the gender-related ideal-types previously identified. In one case, a recruiter from a public administration body stipulated that she, personally, did not hire men for apprenticeships positions because she felt more at ease with women:

“*Well, how do we hire? That’s a tricky question. We used age criteria because that was the easiest, so we took not too old and not too young, for different reasons. I did eliminate men, because I always wanted to have women (laughs).*”

Recruiter, Public Administration, female)

Here again, we can see how personal preferences could affect the apprentice’s selected profile and introduce a gender bias. On the other hand, another recruiter underlined how unexpected it was to see a woman able to manage a team of men when he described the role of a former apprentice he hired:

“*Girls? No problem. Girls that come here, who are ready to undertake this profession, are better than men when it comes to leading a project. Because I push them to develop authority, one has to show their teeth without being aggressive. I remember two apprentices, they were managing these men very well. And in order to do so they needed to be extremely competent, as much as men, that goes without saying, but with an additional skill that is natural authority. This becomes increasingly important (…) and we were saying, “that’s incredible, those girls, they are 22, 23 years old and they manage projects with 10, 15 tradesmen and at least as many workers.*”

Recruiter, Carpentry Company, male)

Stereotypes came into play in the recruiters’ mind maps. Similarly, picturing the 3-day training course that was used to select him as a future apprentice, a student explained what played in his favor:

“*During the last 3 years, they only had girls as apprentices so when I arrived the manager said, “At last, a boy!” Indeed, there are often heavy things to carry so the fact that I was a boy has played a positive role, so yes, physical abilities.*”

Apprentice, seller, male 18 years old)

Soft skills and “feeling” appeared to constitute two important dimensions of the hidden curriculum. They consisted of assessing the candidate’s potential “fit” with the company and their immediate working environment. Besides these interaction-related skills, other dimensions appear at act that account for additional skills and abilities in the hidden curriculum: anticipation, autonomy, and reflexivity.

###### Anticipation, autonomy, and reflexivity

Interviews underlined the importance of being able to anticipate, demonstrating autonomy and displaying some reflexivity, i.e., the ability to give a sense to one’s apprenticeship plans and to demonstrate it in order to be recruited.

As previously highlighted by the future apprentices, building up to the recruitment process begins in the second year of the lower (I) secondary cycle, with a compulsory 1-week internship which they organize themselves. This internship is the first opportunity to consider the type of profession and work environment one might choose, but also to develop professional contacts, which could help nurture the students’ social capital. For recruiters, the time-related nature of the recruitment process varies according to the company’s size and organization, but could last almost a year. This lengthy process, as well as the student’s ability to manage it, generated a second discrimination, notably among students having linear trajectories. Indeed, anticipating and building their career plans through internships constituted a tool to convince a potential recruiter.

“*Sometimes there are people who ask very, very early. Sometimes even 1 or 2 years in advance. I remember having people who asked me very, very early. Well they often are right. Since I was able to carry out interviews, internships in the long run, and to confirm that they actually were very motivated people. Often with people that come late to me, like June etc., it is a bit like a safety net. They haven’t really thought about it.*”

Recruiter, Carpentry Company, male)

The ability to anticipate was then perceived by recruiters as a guarantee of the future apprentice’s motivation, coupled with their ability to differentiate themself in the recruitment process:

“*I often conduct workshops in the careers showroom (…). First we hold a conference on how to look for an apprenticeship position, then we organize job interview simulations where young people can come and present their application form to us, etc. We also give out our business cards there, like that, as well as receiving some applications. So it’s also a lot of word-of-mouth (…). So, when there are young people coming to the end of a contract then yeah, for sure. When there’s a nice feeling with the young people who took time to come in the careers showroom that means they are interested, if they took the time… Well, me in general, I am there on Sunday, so the one who’s coming on Sunday afternoon to have an interview, means they are interested, so yeah, then it can lead to contracts in the end.*”

Recruiter, Human Resources manager, Insurances Company, female)

Two dimensions linked to this relationship with time appeared to structure the recruitment process and they attested to specific recruiters’ expectations. Thus, when asked about “good criteria” influencing apprentice selection, a recruiter answered: “*Those two criteria, I think are really autonomy and motivation.*” Displaying this motivation is expected in the recruitment process:

“*It goes down even better if it is the young person who calls, because we are saying to ourselves,”they are motivated, they didn’t send their application for nothing. So, in this case if the young person calls and then asks for the status of their application, they are reiterating their motivation on the phone. In this case, we’re taking this into account.*”

“*The first thing we judge is how much motivation they have. And why they apply here. Often, the parents call at some point during the recruitment process, but, we are more attentive if the young person calls. Because we say to ourselves, look they are calling. The perception is better because we then think, well, they are motivated and they did not send their application for nothing. So when the young person calls, we take it into account.*”

Recruiter, public administration, male)

“*Well, receiving an application from a student currently on a ‘transition year,’ it’s not the same if they do it in September or in April. What’s critical is that they have to target what they really want to do. We don’t have time to lose with people who look for an apprenticeship and not a profession.” (…) “It means that they have to… that they can’t just produce a simple file as usual, they must contact us by phone, explain their situation. But just a file, here, is rejected.*”

Recruiter, Electricity Company, male)

Finally, according to the recruiters, the ability to anticipate and be active was linked to the apprenticeship candidate’s degree of autonomy and motivation. Apart from academic qualities, the ability to obtain an apprenticeship position thus depended on the students’ ability to justify their actions and choices and to demonstrate their reflexivity.

A 21 year-old female, first year commerce apprentice was invited to recall the questions she was asked during the interview. She answered:

“*Yes, I remember one question that really affected me, it was, ‘Why you and not someone else?’ As I said before, it is clearly this type of question where one has to find a balance. We cannot put ourselves forward too much; we have to show some confidence in ourselves, but not too much either.*”

Apprentice, commercial employee, female, 21 years old)

This is echoed in this recruiter’s comments:

“*Through the testing, we want to know more about their personality, not what they learned at school but if they are able to translate, what they learned at school into what we are asking them to do…*” *(…) “It’s really a question of perception. I try to understand their motivations…*”

Recruiter, carpentry company, male)

Far from being specific to the context under study, this exhortation for autonomy, appeared to be part of a standard framework characterizing democratic societies, and can thus be analyzed as a new heteronomy (Ehrenberg, 2010). As underlined by A. Ehrenberg, during the last decades of the 20th century, “The ability to affirm oneself in an appropriated and controlled way becomes a central ingredient of the socialization process at all levels of social hierarchy,” and still, autonomy “depends on its social conditions.” More generally, the apprentice recruitment process illustrated how, “in the displacement from the entitled to the possible, personal assertion and self-affirmation, are at the heart of democratic society” (Ehrenberg, 2010, p. 13). But, what are the consequences, if “we are now trained since our early childhood to become ourselves” (Ehrenberg, *ibid*)? This concept of autonomy has two sides. It is both about “freedom of choice on behalf of self-ownership,” as illustrated by the apprentices’ ability to choose their apprenticeship subject area, and by the “ability to act on one’s own in most everyday life situations,” which we depicted as a key asset in the recruitment process.

Autonomy, vocation, and “practical sense” appeared to be keywords to access apprenticeships. The exhortation for autonomy that was made to the apprenticeship applicants in the recruitment process appeared to be an implicit rule. This rule operated in different ways. For example, in the recruiters’ search for “authenticity”:

“*The aim is really to have an authentic person in front of us, which is linked to what they are saying and the appearance they give.*”

Recruiter, shop manager, large-scale distribution, male)

Although these informal requirements helped build the profile of the recruited apprentices, their effects were not unilateral, as the two following interview extracts show:

“*On one hand, yes we can help them, we do also have young people who have family situations that aren’t easy so I think we can provide them with something. It is also a criteria. We tell ourselves, “Maybe they will need us.” There have been times when we didn’t recruit someone who had more “chances” and instead we took someone who needs us more and with who we can be more tolerant and more understanding than a private company, for example. So we do try to give those young people opportunities.*”

Recruiter, public administration, female)

“*He was a disabled young person. We took him for the apprenticeship. We should never have employed him under normal conditions. He insisted so much and was so prompt to undertake this apprenticeship that he did it. (…) So we have to be able to listen to people, their desires and to see their motivation and especially their authenticity. If they are respectful, they can go far. (…) So, we have to reach out to others from time to time, even if it is difficult sometimes. We take it on ourselves and we have to be able to go this way, (…) to help out.*”

Recruiter, shop manager, large-scale distribution, male)

These comments highlight the fact that the exhortation for autonomy in the recruitment process periodically applied to recruiters and provided some flexibility in the use of informal norms. For the apprentices, overcoming conventional recruitment criteria giving some sense to the recruiter’s activity by replacing it with their personal relationship to the world (in a specific ethos), appeared to be the required counterpart of autonomy. Finally, and schematically, the apprentice recruitment process could then be seen as the meeting of those two types of autonomy. The three following interview extracts revealed this connection between the recruiters’ and the apprentices’ autonomy:

“*I think that, principally, the young have to look for something they really like, and go all-out for it. It takes a lot of energy but they have to show real interest, that’s crucial. That’s what we realize the most, that people come here because they were pushed to do so… We can show them the job, but if they don’t have some get up and go in them… They have to build their training period, they have to try to… They must not just arrive, they have to show a minimum of interest.*”

Recruiter, Electricity Company, man)

“*It’s like a young bird flying the nest. It’s exactly the same thing. Yet, our job is to be instructive, to be able to give ourselves time because today, in the economic context we have, we want everything right away, and it’s not always easy because there are two contradictory dimensions. These are the fact that we tell ourselves we will give ourselves time, but we have less and less time, and the second issue is that, financially, we are always restricted by our costs.*”

Recruiter, Retail Company, male)

“*I knew they started recruiting on the 1st of March, so on the 1st of March I went to the post office in order to have a stamp with the date on it, so that they see my interest, so that they see my commitment.*”

Apprentice, bookseller, female, 24 years old)

## Discussion

In this study, we have examined how students access apprenticeships and how they valued an apprenticeship. In a nutshell, this research leads us to distinguish between three processes that appeared important and cumulative in the recruitment process.

Firstly, we found that conventional dimensions known to affect access such as schooling trajectories, family support, and social resources combined in different trajectory configurations leading to varying degrees of difficulty in accessing an apprenticeship.

Secondly, our research highlighted the additional role played by a hidden curriculum in finding an apprenticeship. Its content was identified and it was revealed how this hidden curriculum lead to promoting a specific type of individual. This hidden curriculum mainly came into play when the candidate was assessed *in situ* through a preliminary internship or an interview, and more generally through all types of interactions involved in the recruitment process. The hidden curriculum comprises several dimensions: such as anticipation, autonomy, and reflexivity. Those selection practices based on the possessing of a specific type of soft-skill appeared to penalize young people with specific trajectory configurations as characterized by ideal-types 3 and 4 in our analysis. These included students with non-linear schooling trajectories in the lower or medium academic stream who used transition measures, had little family support and/or social capital, as well as students with linear schooling trajectories in the weakest academic streams with no family support. These students faced greater difficulties in dealing with the hidden curriculum as they could not demonstrate the required aptitudes. Indeed, these aptitudes are more likely to come from primary socialization than from secondary socialization backgrounds, particularly when performed within compulsory schooling. This leads us to concur with Lahire (2014, GRS website) in that, “What determines the activation of one disposition in a specific context is the product of the interaction between internal and external power relations: power relations between aptitudes that are more or less built during past socialization (internal) and power relations between elements (such as the situation, objectives, and characteristics that can be associated with different people) of context that more or less weighs on the actor (external).” Therefore, previous socialization influences the recruitment situation, as in this context, the (non) activation of some “aptitudes” influences the recruiter’s choice. Consequently, the hidden curriculum used to identify the “right apprentice” tends to (re)produce inequalities between students depending on their school results as well as their social and cultural skills and their ability to anticipate and act autonomously. This exhortation for autonomy corresponds to the search for a specific type of individual that the recruitment process makes perform as a “subject.” This is defined by Touraine (1995, p. 29) as “…the desire to be an individual, to create a personal history, to make sense of all the experiences of individual life…” Indeed, “the Subject is neither the individual, nor the self, but the work through which an individual transforms into an actor, meaning an agent able to transform their situation instead of reproducing it.” (Touraine, 1992, p. 476). In the end, the “right” apprentice appears to be the subject of their recruitment search. Still, the use of a hidden curriculum and the lack of collective preparedness in the recruitment process, leaving the student alone with their own resources, transform this recruitment criteria into a social reproduction tool. By asking a young students to present themself as the subject of their recruitment – without providing them with collective resources aimed at helping them take a step back and nurturing their reflexivity – leads to promoting the ability to present oneself as a subject, as a social aptitude and not as a universal right as expected in democratic societies.

Thirdly, being able to present oneself as a subject in the recruitment process appears necessary but not always sufficient to easily access apprenticeships. Ultimately, if the process of subjectification is assessed in apprentice recruitment, aptitudes, and social characteristics can influence hiring both ways, depending on how apprentices interact with the recruiter’s own aptitudes and perception of their role as a recruiter, for their company, but also more generally in their society. Only by taking account of these three processes and how they are interlinked can one fully understand institutional barriers to equal opportunities in educational trajectories.

## Conflict of Interest Statement

The authors declare that the research was conducted in the absence of any commercial or financial relationships that could be construed as a potential conflict of interest.

## Footnotes

1 All interviews were carried out in the frame of collective research by 23 students who were involved in our sociology of education seminar. We want to thank them for their investment in this project.

2 The Organism for scholar Improvement, Transition and professional Insertion (OPTI) is a transitional measure receiving young people between 15 and 18 years old requiring an additional year to find their way.

3 In their book, *De la justification*: Boltanski and Thévenot (1991) identified different worlds controlling organizations in terms of conventions and agreements. Each of those different worlds refers to values, characteristics, and (un)enhanced subjects within them. For an introduction to those worlds, see the chapter of Imdorf and Seiterle (2015) that articulates inequalities faced by young people of immigrant descent at the beginning of an apprenticeship, with the nomenclature of certain worlds encountered in different companies.
